# Elastic stable intramedullary nailing for severely displaced distal tibial fractures in children

**DOI:** 10.1097/MD.0000000000004980

**Published:** 2016-09-30

**Authors:** Kaiying Shen, Haiqing Cai, Zhigang Wang, Yunlan Xu

**Affiliations:** Department of Pediatric Orthopedics, Shanghai Children's Medical Center, affiliated to Shanghai Jiaotong University School of Medicine, Shanghai, China.

**Keywords:** children, close reduction, distal tibial fracture, elastic stable intramedullary nail

## Abstract

Elastic stable intramedullary nailing (ESIN) has became a well-accepted method of osteosynthesis of diaphyseal fractures in the skeletally immature patient for many advantages, the purpose of this study is to evaluate the preliminary results of this minimally invasive treatment for severely displaced distal tibial diaphyseal metaphyseal junction (DTDMJ) fractures.

This study was carried out over a 6-year period. Twenty-one severely displaced DTDMJ fractures treated using ESIN were evaluated clinically and radiographically. Complications were assessed: the patients were evaluated with regard to nonunion, malunion, infection, growth arrest, leg length discrepancy, implant irritation, and joint function.

Mean age at the time of surgery was 7.8 years (range between 5.3 and 14.8 years), mean body weight 34.1 kg, all fractures were transverse or mild oblique type, including 3 open fractures, 5 multifragmented fractures, and 4 fractures associated with polytrauma; 6 cases were treated with antegrade ESIN of tibia while 15 cases need combined retrograde fibula and antegrade tibia fixation treatments. Follow-ups were ranging from 11 to 36 months, 19 fractures showed both clinical and radiographic evidence of healing within 5 months; all cases had full range motion of knee and ankle with symmetrical foot progress angle. Nail removal was at a mean 7.1 months, at final follow-up, no growth arrest or disturbances occurred. Five patients had complications; leg length discrepancy had decreased yet affected 2 patients, 2 cases showed delayed union, and 1 case developed restricted dorsal extension at the metatarsophalangeal joint of the hallux.

ESIN is the treatment of choice for pediatric severely displaced DTDMJ fractures that cannot be reduced by closed reduction or ones that cannot be casted. The advantages include faster fracture healing, excellent functional and cosmetic results, safe and reliable surgical technique, and lower severe complication rate.

## 1 Introduction

Distal tibial metaphyseal fractures in children are usually treated without surgery by close reduction and casting during 6 to 8 weeks. Surgical treatment should be considered for those cases where closed treatment cannot achieve and maintain acceptable alignment, rotation, and length. As severely displaced distal tibial diaphyseal metaphyseal junction (DTDMJ) fractures, the treatment is more challenging, and often requires intervention, operative treatment is needed in polytrauma patients who have most unstable fractures, open fractures, neurovascular injuries, and compartment syndrome.

Tibial diaphyseal displaced fractures in children are frequently treated with the elastic stable intramedullary nailing (ESIN) method. The advantages are minimally invasive surgery with a short hospitalization duration, primary bone union and early weight bearing.^[1–4]^ ESIN can be considered the “gold standard” for the treatment of midshaft fractures, but there are concerns about their use in distal tibial diaphyseal and proximal metaphyseal fractures, its proximity to the ankle makes the surgical technique more complicated than the treatment of the midshaft fractures, and the stability supplied by the nails cannot be achieved optimally.

Due to the paucity of the relevant literature to investigate the outcomes of surgical treatment of severely displaced DTDMJ fractures using ESIN in children, we report our initial experience and retrospectively evaluate the clinical and radiographic outcomes.

## 2 Materials and methods

**Figure 1:**
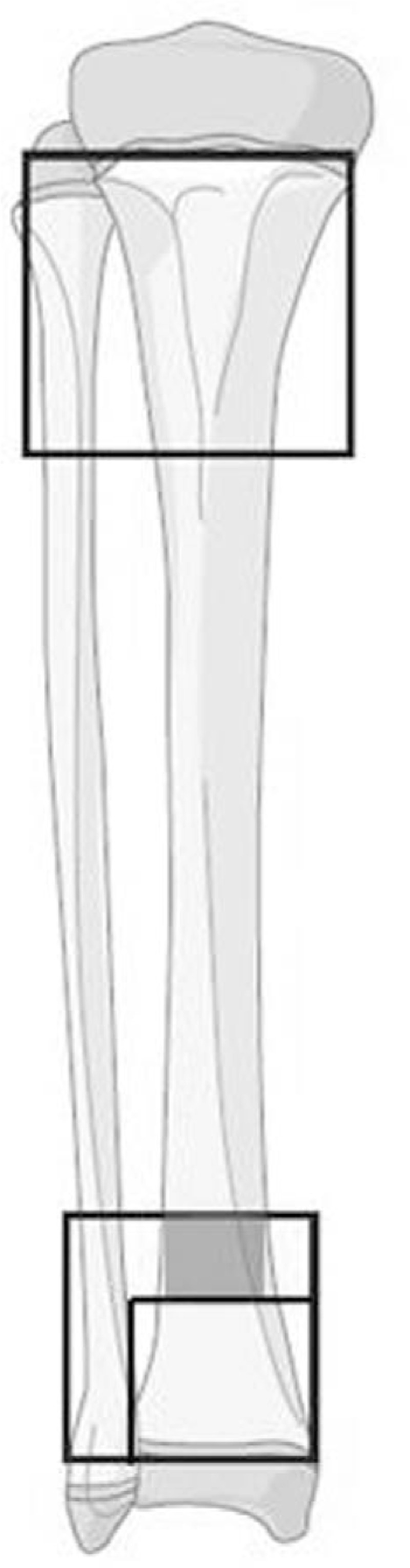
The segment of distal tibia between 2 squares is defined as the distal tibial diaphyseal metaphyseal junction (the area bounded by the 2 squares in gray).

DTDMJ is defined on the basis of “The AO Pediatric Comprehensive Classification of Long Bone Fractures,”^[5,6]^ the metaphysis area is marked by a rectangle containing the base of the growth plate of both distal tibia and fibula (the big square at the distal tibia and fibula), another small square is made at the side of tibial epiphysis that the height was equal to the widest portion of the tibial epiphysis.^[7]^ The segment of distal tibia between 2 squares is defined as the DTDMJ (Fig. 1, the red area).

### 2.1 Clinical and radiographs evaluation

We retrospectively reviewed the medical records and radiographs of 21 severely displaced DTDMJ fracture without associated neurovascular injuries. Surgery was indicated after failed conservative management, including close reduction and ESIN internal fixation at our institution between 2008 and 2013. All fractures were extra-articular fractures and none of the fractures was pathologic or neuromuscular disorder. Informed consent was obtained and institutional review board approval was acquired.

Plain anteroposterior and lateral radiographs of the full tibia were obtained to assess the location and displacement of the DTDMJ fractures, as well as whether there was a concomitant fracture of the fibula, the mild oblique fracture was defined when the length of the fracture line was less than twice the width of the bone.^[8]^ In this series, all fractures were severely displaced and unstable, tibial fractures were displaced more than two-thirds of the diameter and/or had angulation >30° after manipulation.

At the follow-up, radiographs were assessed by a trained reviewer but not involving in the patients’ care. All cases were assessed with full leg AP and lateral radiographs. Also, a standing weight-bearing position was recommended after fracture healing to assess lower limb alignment and discrepancy. Union was defined as healing of at least 3 of 4 cortices on biplanar radiographs; delayed union was defined as having 1 or 2 cortices healing within 6 months and the union achieved during 7 to 12 months. Nonunion was defined as the lack of any healing within 6 months. Malunion was defined as angular deformity of greater than 4° in coronal plane or sagittal plane, and limb-length inequality ≥1 cm were considered complications.

At the follow-up, physical examination was used to evaluate the knee and ankle ranges of motion and foot progress angle. Malrotation was clinically assessed by the thigh foot angle and to document eventual complications.

### 2.2 Surgical treatment

The patients underwent general anesthesia in the supine position. Titanium elastic intramedullary nails (Synthes TM, Synthes Biomaterials, Swiss) with 2.5 to 4.0 mm diameters were employed on the basis of one-third of the medullary canal of the tibial shaft at its narrowest point. The tips of the nails (the apex at 3–6 cm proximal part to the tip) may need to be greater precontoured approximately 1.3 times as it does in shaft fracture, for more divergence can be achieved with more stabilization of the fracture when nails are advanced into the distal fragment.

**Figure 2:**
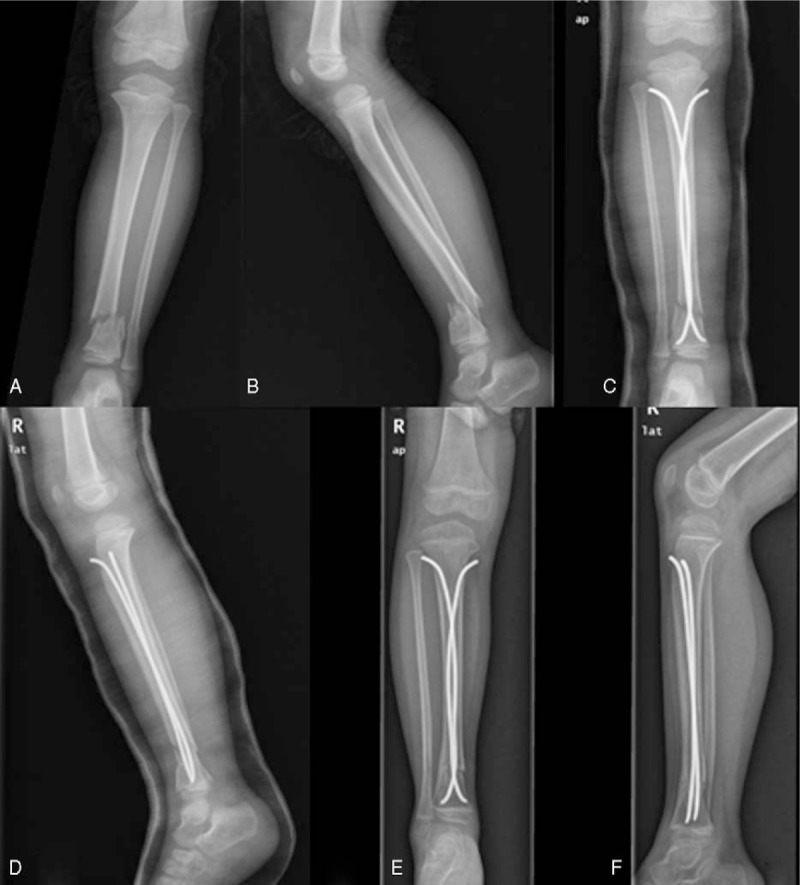
A 6.1-year-old girl fell down from 1.5 m height. A, B, Anteroposterior and lateral views of the severe right DTDMJ fracture, mild oblique type. C, D, Immediate postoperative radiographs after closed reduction and fixation with 2 nails. E, F, 3 months after surgery, the fracture showed radiographic evidence of healing. DTDMJ = distal tibial diaphyseal metaphyseal junction.

**Figure 3:**
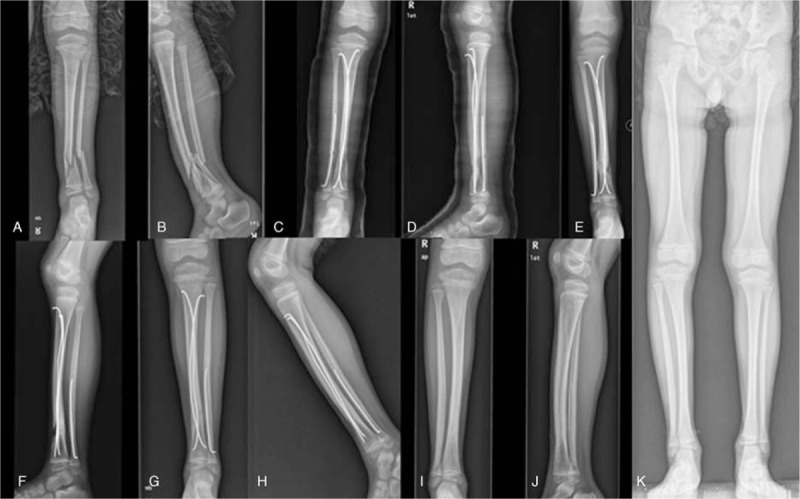
A 7.4-year-old boy suffered traffic accident. A, B, Anteroposterior and lateral views of the severe right oblique DTDMJ fracture, multifragmented open fracture, Gustilo type I. C, D, Tibia was fixed with 2 nails, fibula with 1 nail. E, F, 6 months after surgery, the fracture with radiographic evidence of delay union. G, H, 10 months after surgery, the fracture showed evidence of healing. I, J, 16 months after surgery, no growth arrest or disturbances by radiological assessment. K, 24 months after surgery, standing weight-bearing view showed 10 mm overgrowth on the right leg.

**Figure 4:**
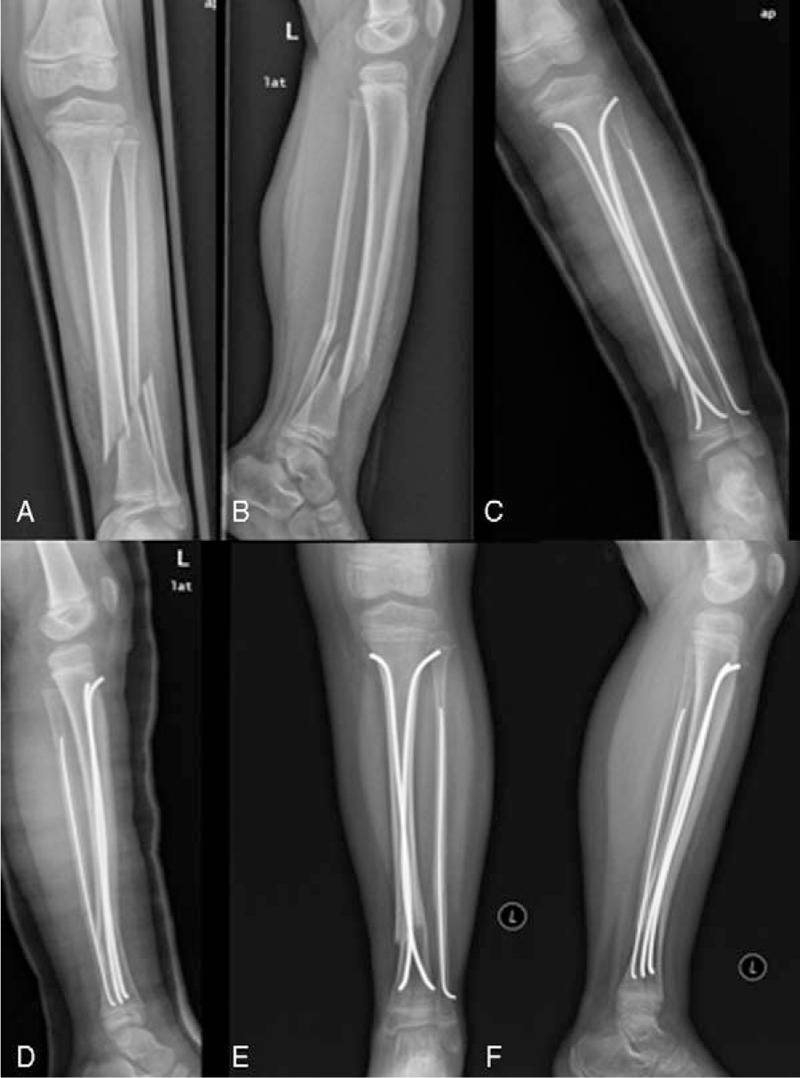
A 7.1-year-old boy suffered traffic accident. A, B, Anteroposterior and lateral radiographs of the left leg with mild oblique unstable DTDMJ fracture. C, D, Immediate postoperative radiographs after closed reduction and fixation with ESINs. E, F, 6 months after surgery, the fracture showed radiographic evidence of healing.

**Figure 5:**
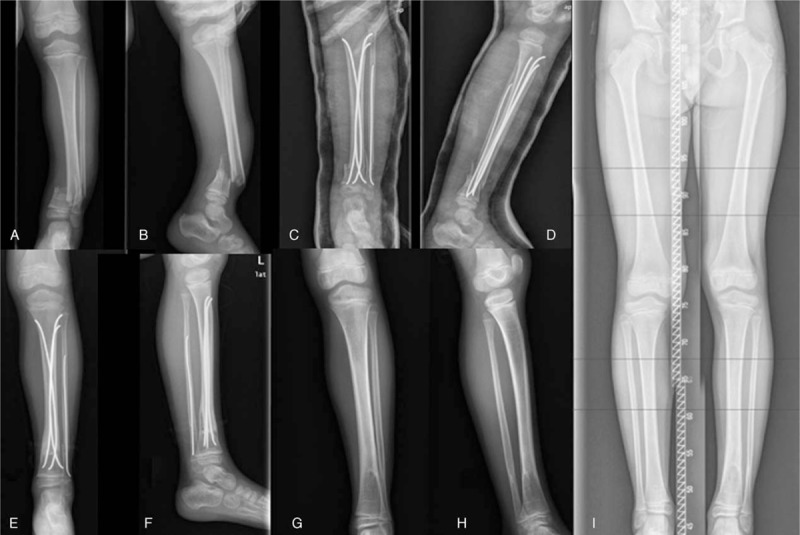
A 5.5-year-old boy suffered traffic accident. A, B, Anteroposterior and lateral views of the severe left DTDMJ fracture, multifragmented open fractures Gustilo type I. C, D, Immediate postoperative radiographs after closed reduction and fixation with ESINs, tibia was fixed with 3 nails, fibula with 1 nail. E, F, 5 months after surgery, the fracture showed radiographic evidence of healing. G–I, 36 months after surgery, no growth arrest or disturbances by radiological assessment, standing weight-bearing view showed 11 mm overgrowth on the left leg. DTDMJ = distal tibial diaphyseal metaphyseal junction, ESIN = elastic stable intramedullary nailing.

The surgical technique was based on that described by Me’taizeau and Ligier.^[1]^ At the beginning, 2 nails were introduced through symmetrical skin incisions (1 cm in length) which were made at the proximal medial and lateral metaphyseal cortices 2 cm distal to the tibial tubercle, and both nails were advanced proximally to the fracture site; then close reduction of the fracture was performed using C-arm fluoroscopy, distal traction was continued until length has been reestablished; after that, distal fragment was manipulated to contact with proximal one. Once a satisfactory reduction has been achieved, the first nail was advanced distally into the tibal metaphysis (distal fragment). The position of distal fragment could be corrected indirectly by turning the first nail, and then the second nail was advanced into the proper position. Make sure that the tips of nails were in divergent positions and the reduction and stability of the fracture were satisfactory (Figs. 2–4); otherwise, an extra nail will be inserted to get more stabilization of the fracture (Fig. 5). The third entrance site was made 1 cm distally and anteriorly to the lateral one; all the tips should not be perforated at the growth plate. Finally, if the fibular fracture cannot be reduced acceptably followed the tibial fracture's reduction, 1 nail of adequate size was inserted from distal to proximal with the insertion point above the physis (Figs. 3–5). All patients, after surgery, were immobilized in full-leg casts for 2 weeks and changed to below knee casts for 4 weeks, and weight bearing was allowed progressively after cast removal while union was defined on radiographs.

None of the fractures needed open reduction and the debridement was performed before reduction in open fractures.

## 3 Results

There were 11 boys and 10 girls. And the mean age at the time of their surgeries was 7.8 (range, 5.3–14.8) years, mean body weight 34.1 (range, 23–58.5) kg, in which 9 cases involved the left side. The main cause of injury was traffic accidents in 10 cases, by tumbling in 6 cases, and by falling from height in 5 cases. The most common was the transverse type of fracture (13 cases) followed by the mild oblique type (8 cases); 4 multifragmented fractures were classified as comminuted type “wedge” while 1 multifragmented fracture was “complex” based on AO's definition of fracture severity code and proposed simplification;^[6]^ 3 cases had mild open fractures (Gustilo type I), and 4 cases of polytrauma included 3 multiple fractures and 1 had craniocerebral injury. In the series, all cases were associated with distal fibular fractures, from which 18 fibular fractures were completely displaced.

In tibial fractures, 19 cases were fixed with 2 nails (Figs. 2–4), while 2 fractures were fixed with 3 nails to stabilize the fragments (Fig. 5); there were 5 cases with nails size 2.5 mm in diameter (in total 11 nails, 1of 5 cases was fixed with 3 nails), 14 fractures by 2 nails size 3 mm in diameter (28 nails), 2 fractures by 3.5 mm nails (in total 5 nails, 1 of 2 cases was fixed with 3 nails). In fibula fractures, 15 of 21 cases were retrograde fixed with ESINs (Figs. 3–5), 10 fractures by 1 nails size 2 mm in diameter and 5 cases by 1 nail size 2.5 mm.

Mean duration of surgery was 46.7 ± 20.0 (range, 25–85) minutes. Mean hospitalization duration was 3.9 (range 2–10) days; it did not exceed 5 days except in the case of the patient with craniocerebral injury. Follow-up ranged from 11 to 36 months, averaged 24.6 ± 8.5 months, 19 fractures showed both clinical and radiographic evidence of healing within 21 weeks (mean, 9.6 weeks; range, 8–21 weeks) and 2 cases (9.5%) showed delayed healing at 10 months (Fig. 3). No angular malalignment or malrotation was identified in all cases; average angulation on coronal plane is 1.9 ± 1.2°, while that on sagittal plane is 1.3 ± 1.3°.

Nail removal was at a mean 7.1 (range, 5–13) months. At the final follow-up, all cases had full range motion on knee and ankle with symmetrical foot progress angle, no limping and they were able to have unrestricted physical activity at the finial visit. There were no infections or nail end irritation, as no growth arrest or disturbances by radiological and clinical assessment.

**Figure 6:**
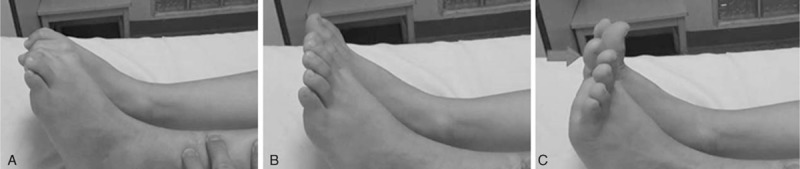
A 5.5-year-old boy with severely displaced DTDMJ fracture on the left side (open fracture Gustilo type I), who developed restriction of the hallux dorsal extension. A, B, 36 months after surgery, when the ankles were passively plantar flexed or in relaxed position, it showed symmetrical plantar flexion at the metatarsophalangeal joint of the hallux. C, When the ankles were dorsiflexed, it showed restricted dorsal extension at the metatarsophalangeal joint of the left hallux (gray arrow).

Five patients had complications. Leg length discrepancy affected 2 cases, initially, lengthening was observed in 2 patients (12 mm, 14 mm) at 12 months after surgery. At the final visit (24 months and 36 months after surgery), the length discrepancy had decreased, and mean tibia overgrowth was10.5 mm (10 mm, 11 mm) (Figs. 3 and 5). Two cases showed delayed healing at10 months after surgery, and they both suffered comminuted fractures. One case developed restriction of the hallux dorsal extension and did not relieve it at 36 months after the surgery, which showed dorsal extension restriction at metatarsophalangeal joint of the hallux when the ankle was dorsiflexed (Fig. 6).

## 4 Discussion

Distal tibial metaphyseal fractures occur frequently. Conservative treatments with immobilization and surgical stabilization by external fixation, plate, or percutaneous pinning have been proposed as possible management options.^[9–14]^ Although reasonable success has been reported with various treatment modalities, most of these cases in children require nonoperative treatment. However, fractures in polytrauma, severely displaced or unstable fractures may benefit from surgical treatment.

Operative methods include close reduction with percutaneous pinning, open reduction and internal fixation with compression plate, external fixator, or minimally invasive plate osteosynthesis (MIPO). However, the optimal treatment of unstable distal tibia metaphyseal fractures remains controversial. Crossed Kirschner wires fixed with an acute angle or entry point are very close to the fracture line, and this would lead to unstable fracture reduction. Plates may sometimes result in extensive soft tissue dissection and may be associated with nonunion, wound infections despite that they could bring complete anatomical reduction. As external fixation provides a stable immobilization, which allows direct surveillance of the limb and wound status. It is particularly useful in open fractures, some studies reported that the use of external fixator was associated with 50% of superficial pin tract infections, a high rate of refracture and joint stiffness.^[15–17]^ MIPO is to preserve the soft tissues at the fracture site while providing adequate stabilization of the fracture; recently, the excellent results in adults^[18–20]^ have supported the treatment of selected distal tibia fractures in a pediatric group.^[14]^ However, patients with a plate-to-physis distance of 20 mm or less in distal femoral fractures were at a significantly higher risk of developing distal femoral valgus deformity;^[21]^ although distal tibial grows slower than distal femoral physis, this also could be a potential complication and requires close monitoring, thus MIPO is recommended in adolescent.^[14]^

In spite of the internal fixation of pediatric diaphyseal tibial fractures with ESIN has been a rapid, well-codified, and effective method.^[2]^ The use of ESIN in the proximal and distal tibia is technically demanding^[3]^ and not free of complications. Only few reports about its application in the distal tibial metaphyseal fracture were found. In 2014, Cravino et al^[22]^ showed good results in the pediatric population who had sustained closed traumatic, displaced fracture of the distal tibial metaphysis treated by ESIN. We separated distal tibial metaphysis into 2 parts, proximal part and distal part. DTDMJ is the proximal part of the distal tibial metaphysis. All the 21 cases in this study were located at this junction, and satisfied results have been achieved. If the fracture is located at the distal part, which is more adjacent to the epiphyseal plate, in our opinion, it will be too difficult to fix the fracture and keep its stabilization by the nails unless the tips of the nails are perforated at the growth plate. The reason is that the fracture line is very close to the growth plate and nails are insufficient for keeping the reduction while the nails stop at the growth plate. Cravino et al^[22]^ reported in some distal tibia fractures, for example, stable fixation could only be achieved by anchoring the tip of the nails in the epiphysis; however, these cases just had short follow-up and it is not possible to exclude growth plate disturbances. Due to the high risk of growth disturbances, in this study, all fractures were located at DTDMJ and none of the nails advanced across the distal epiphyseal plate.

Treatment of the DTDMJ fractures with ESIN involves a totally different concept from diaphyseal fracture management. Although the Me’taizeau technique is minimally invasive and easy to learn, distal tibia metaphysis fractures can be challenging due to the relatively short length of the distal fragment and the proximity of the growth plate. First, the tips of the nails may need to be greater precontoured approximately 1.3 times as it does in shaft fracture to get much divergency with stable osteosynthesis. Secondly, if conventional 2 nails cannot achieve sufficient stabilization of reduction, an extra nail could be used to get more stabilization of the fracture, and all the 3 tips of the nails should be in divergent positions. In this study, 2 of 21 cases fixed with 3 nails in tibial fracture, and 1 case showed in Fig. 5, which was classified as comminuted type “wedge”; another was a 14.8-year-old boy had an unstable fracture fall down from stairs who suffered complex comminuted fracture. Thirdly, all the tips of the nails should stop at the growth plate, which lies immediately above the epiphyseal plate to get more immobilization. Therefore, the ends of the nails should be managed critically precise. Lastly, 2 weeks full-leg cast and 4 weeks below knee cast are recommended to ensure the rigid fixation.

In this series, 19 fractures achieved bony union within 21 weeks and 2 cases showed delayed healing at 10 months after surgery, no angular malalignment or malrotation was identified. This technique is to preserve the blood supply and soft tissues at the fracture site while providing adequate stabilization of the fracture. It included 2 cases with 3 nails fixation of tibia and 15 cases with retrograde fixation of the fibula. Other complications were 2 cases of overgrowth (10 mm, 11 mm) and 1 case developed restricted dorsal extension at the metatarsophalangeal joint of the hallux, 1 study^[23]^ reported 5 patients developed clawing of the hallux following a fracture of the tibia, and relieved by performing a tenolysis proximal to the medial malleolus, the operative findings demonstrated that the etiology was associated with a localized subclinical compartment syndrome. Our patient refused to accept the further treatment because he did not have any limitation during physical activity.

Limitations of this study include a retrospective evaluation of surgical treatment with a small number of patients, the absence of long-term follow-up to evaluate the outcome of growth plate disturbances, and lack of comparison group to carry out randomized controlled trials.

## 5 Conclusion

This study showed good functional and radiological results in the pediatric population who had severely displaced DTDMJ fractures that cannot be reduced by closed reduction or be casted, and we believe that ESIN is the treatment of choice for the transverse and mild oblique fractures, “wedge” multifragmented fractures, and mild open fractures. The advantages include fracture healing faster, excellent functional and cosmetic results, safe and reliable surgical technique, and lower severe complication rate. However, further prospective studies are required to compare ESIN with other treatment modalities.
